# High-Performance Thin-Layer Chromatography-Immunostaining as a Technique for the Characterization of Whey Protein Enrichment in Edam Cheese

**DOI:** 10.3390/foods11040534

**Published:** 2022-02-12

**Authors:** Mascha Treblin, Tobias von Oesen, Jana Lüneburg, Ingrid Clawin-Rädecker, Dierk Martin, Katrin Schrader, Ralf Zink, Wolfgang Hoffmann, Jan Fritsche, Sascha Rohn

**Affiliations:** 1 Institute of Food Chemistry, Hamburg School of Food Science, University of Hamburg, Grindelallee 117, D-20146 Hamburg, Germany; mascha.treblin@chemie.uni-hamburg.de (M.T.); jlueneburg@gmx.net (J.L.); 2 Department of Safety and Quality of Milk and Fish Products, Federal Research Institute of Nutrition and Food, Max Rubner-Institut, Hermann-Weigmann-Straße 1, D-24103 Kiel, Germany; tobias.vonoesen@mri.bund.de (T.v.O.); ingrid.clawin-raedecker@mri.bund.de (I.C.-R.); dierk.martin@mri.bund.de (D.M.); katrin.schrader@mri.bund.de (K.S.); wolfgang.hoffmann@mri-bund.de (W.H.); jan.fritsche@mri.bund.de (J.F.); 3 Center of Expertise Research & Technology (CoE-R&T), DMK Group (Deutsches Milchkontor GmbH), Flug-Hafenallee 17, D-28199 Bremen, Germany; ralf.zink@dmk.de; 4 Department of Food Chemistry and Analysis, Institute of Food Technology and Food Chemistry, Technische Universität Berlin, TIB 4/3-1, Gustav-Meyer-Allee 25, D-13355 Berlin, Germany

**Keywords:** whey protein-fortified cheese, HPTLC immunostaining, cheese ripening, tryptic digestion, epitopes, β-lactoglobulin

## Abstract

Whey protein-enriched cheese can be produced by means of a high-temperature treatment of a part of the cheese milk. In this way, the nutritional quality of the resulting cheeses can be increased while resources are conserved. High-performance thin-layer chromatography-immunostaining (HPTLC-IS) using specific β-lactoglobulin (β-LG) antibodies was applied to study the implementation and stability of β-LG in two different sample sets of whey protein-enriched Edam model cheeses, including industrial-scale ones. Two methods were compared for the extraction of the proteins/peptides from the cheese samples. By applying tryptic hydrolysis directly from a suspended cheese sample instead of a supernatant of a centrifuged suspension, a better yield was obtained for the extraction of β-LG. When applying this method, it was found that selected epitopes in the tryptic β-LG peptides remain stable over the ripening period of the cheese. For four of the tryptic β-LG peptides detected by immunostaining, the amino acid sequence was identified using MALDI-TOF-MS/MS. One of the peptides identified was the semi-tryptic peptide VYVEELKPTP. A linear relationship was found between the content of this peptide in cheese and the proportion of high-heated milk in the cheese milk. β-LG enrichment factors of 1.72 (*n* = 3, sample set I) and 1.33 ± 0.19 (*n* = 1, sample set II) were determined for the cheese samples containing 30% high-heated milk compared to the non-enriched samples. The relative β-LG contents in the cheese samples with 30% high-heated milk were calculated to be 4.35% ± 0.39% (sample set I) and 9.11% ± 0.29% (sample set II) using a one-point calibration. It can be concluded that the HPTLC-IS method used is a suitable tool for the analysis of whey protein accumulation in cheese, being therefore potentially directly applicable on an industrial scale. For more accurate quantification of the whey protein content in cheese, an enhanced calibration curve needs to be applied.

## 1. Introduction

In traditional cheese production, whey with its proteins is removed, and only the casein fraction remains in the curd [1,2]. Consequently, whey has traditionally not been used for human nutrition but only for animal feed or has been discarded. Nevertheless, whey is increasingly used in the food industry due to its valuable constituents [1,3,4,5,6].

Whey proteins have a significant biological value due to their high content of cysteine, tryptophan, and branched amino acids. Whey even exceeds the biological value of egg white by about 15% [1,4,5,7]. Its proteins account for about 20% of the total milk proteins, with β-lactoglobulin (β-LG), α-lactalbumin, and bovine serum albumin being the most abundant ones, besides minor proteins such as lactoferrin, immunoglobulins, growth factors, and enzymes [5,7,8]. β-LG is the major whey protein in bovine milk, with a concentration of 3.2 g/L [8,9].

For a more resource-efficient process and an increase in the nutritional value of cheese, modern techniques are developed for potentially incorporating whey proteins into the cheese matrix on an industrial scale [10,11,12]. Thereby, the whey proteins are applied either in their native or in a denatured form [1,10]. They can be retained in their native form by concentrating the milk using ultrafiltration [1,10,13]. For an incorporation of denatured whey proteins, intensified heat treatment of the initial milk can be performed [1,10,11,14]. As a result, thermally induced denaturation of the whey proteins leads to the exposure of reactive thiol groups, which are responsible for the aggregation of the whey proteins with each other or with the caseins through neo-formed disulfide bonds. The whey proteins become precipitable by acid and end up in the cheese matrix [1,3,9,14,15,16]. In addition, whey protein enrichment can be achieved by combining membrane filtration and high-temperature heating, or denatured, particulated whey proteins can be added to the milk or the curd before pressing [10,11,12,14,17].

During the ripening of cheese, proteolysis occurs, which has a significant influence on the texture and the flavor of the final cheese [16,18,19,20]. Many different enzymes from various sources are responsible for this proteolysis, but all result in degradation products such as peptides, amino acids, and volatile organic compounds [13,18,21]. The proteases and peptidases mainly originate endogenously from the initial milk, the enzyme preparations added for casein denaturation, starter culture bacteria, or ubiquitously cultures such as mold and non-lactic acid bacteria [16,18,22]. While chymosin and pepsin are the most common coagulant enzymes, plasmin is the most important endogenous enzyme in milk [18,21]. Cheese ripening can be divided into two phases. In this process, the coagulant is mainly responsible for the first phase of proteolysis, which lasts one to two weeks [13,16,18,19]. During this period, hydrolysis of proteins occurs, leading to the formation of larger to medium oligopeptides [13,18,21]. In contrast, the second phase of cheese ripening is much slower and lasts weeks to months [13]. Over this time, the initial peptides are further hydrolyzed by the proteinases and peptidases of the microorganisms, yielding short peptides and amino acids [16,18,21].

In the production of whey protein-enriched cheese, ripening may be negatively affected. On the one hand, this is due to complexes between β-LG and κ-casein formed via disulfide bonds as described above, so that the accessibility of κ-casein for chymosin is reduced. On the other hand, the activity of plasmin is decreased as the enzyme undergoes complexation with β-LG [1,10,14,16,23].

Being a fermented product, cheese represents a complex matrix for food analysis, as the enzymes and microorganisms have led to various degradation processes of the ingredients such as proteins, carbohydrates, and lipids [24]. As already mentioned, the complex mixture of proteins continuously changes qualitatively and quantitatively during cheese production [22,25]. However, there are many different methods for analyzing the cheese proteome. In most of these methods, the proteins and peptides are fractionated before analysis [18,22,25,26]. This is often followed by two-dimensional gel electrophoresis (2D-GE) and identification of the proteins using tandem mass spectrometry (MS/MS) or, as performed in former times, by Edman degradation [18,22,25,26]. Other detection methods such as Coomassie blue staining for proteins, periodic acid-Schiff base staining for identifying glycoproteins, silver staining for minor proteins, or even immunoblotting are also applicable following 2D-GE separation [22,27,28]. More recently, other separation techniques such as liquid chromatography (LC) or capillary electrophoresis (CE) are used and coupled to mass spectrometry for the analysis of the milk proteome [18,22,25,26].

However, one- or two-dimensional high-performance thin-layer chromatography (1D- or 2D-HPTLC) can serve as an alternative chromatographic technique but has not yet been applied to the analysis of cheese proteins. Especially, novel cheese products, in which whey proteins are enriched, have not yet been analyzed in this way. HPTLC, in combination with different detection methods such as MS, ninhydrin or fluorescamine staining (FS), immunostaining (IS), or aptastaining, has been already successfully applied exemplarily for the analysis of intact and tryptically hydrolyzed milk proteins [29,30,31,32,33,34,35,36,37]. It is a cost-effective method as many samples can be analyzed simultaneously with only small solvent consumption. In addition, various stationary and (gradient) mobile phases as well as detection options are available [38,39,40,41,42,43,44,45,46]. Through the application of fingerprinting, HPTLC can also provide a simple and reliable method for quality control. For example, the quality control of herbal medicinal substances has been performed using HPTLC-fingerprinting for already quite a long time [47].

Compared to GE, HPTLC allows the analysis of peptides with a molecular weight below 3 kDa so that hydrolyzed samples or the proteome of fermented products such as ripened cheese can be analyzed [35,48]. Moreover, effect-directed analysis (EDA) can be applied directly on the same HPTLC plate [35]. This is not the case with electrophoresis, where a transfer of the sequences of interest (e.g., Western blotting) is required prior to EDA [35].

With HPTLC, there is also no loss of analytes (especially posttranslational modifications) during the chromatographic separation, as happening during LC in the pre-column or at the beginning of the separation column [33,40]. With regard to bioactivity, it is not possible to determine the allergenicity or the presence of epitopes of the analytes in high-performance liquid chromatography (HPLC), but using HPTLC-immunostaining (HPTLC-IS) [35]. However, HPLC is a more robust technique compared to HPTLC and is more precise and sensitive in replication and quantification [49,50].

Although being enriched in cheese, the yield of whey proteins is still only low and in a smaller proportion compared to the caseins. Guinee et al. (1995) estimated that whey proteins accounted for 1.6–14.7% of the total protein content in cheeses where the milk was heated differently in terms of temperature and treatment times (72–100 °C/15–120 s) [14]. This makes it difficult to quantify the whey proteins in these whey protein-enriched cheeses, especially when considering the proteolysis during ripening. By coupling HPTLC with IS, it is possible to stain very sensitively and specifically with antibodies against β-LG and its peptides [35,40].

The cheese samples investigated in this work were of the Edam type. Edam belongs to the semi-hard cheeses and ripens for at least four weeks [51]. For this reason, the progression of ripening should be considered, as only ripened products are supplied to the consumer. During the ripening process, proteolysis of proteins occurs. In traditional cheese, this mainly involves the caseins; the degree to which the whey proteins are affected by proteolysis has not yet been adequately clarified [16].

The aim of this work was to evaluate an HPTLC-IS methodology for the analysis of whey proteins and peptides in cheese. The HPTLC-IS method should be applied for the detection of β-LG (and its peptides) by the specific binding of polyclonal antibodies. It should be characterized to which extent the intensive heat treatment of the milk leads to an increase in whey proteins in the cheese. Further, it should be evaluated whether correlations can confirm the linearity of the initial whey protein accumulation or if other mathematical relationships apply. In addition, the ripening-induced degradation of whey proteins was characterized at the hand of samples along the production line from cheese milk to cheese ripened for 13 weeks. Finally, it answers the first questions for a potential industrial roll-out for this very promising methodology.

## 2. Materials and Methods

### 2.1. Materials

#### 2.1.1. Reagents

Acetone, acetic acid, ammonia, ethanol, and methanol (HPLC-grade) were obtained from VWR International GmbH (Darmstadt, Germany). Fluorescamine and iodoacetamide (IAA) were purchased from AppliChem GmbH (Darmstadt, Germany). 2-Butanol, potassium carbonate, potassium dihydrogenphosphate, sodium bicarbonate, sodium carbonate, and sodium chloride (NaCl) were obtained from Grüssing GmbH (Filsum, Germany). Acetonitrile (MS-grade), ammonium bicarbonate, citric acid monohydrate, disodium hydrogen phosphate, dithiotreitol (DTT), isoleucine, 3,3′,5,5′-tetramethylbenzidine (TMB), trifluoroacetic acid (TFA), tris-(hydroxymethyl)aminomethane (Tris), tris-(hydroxymethyl)-aminomethane hydrochloride (Tris-HCl), and urea were purchased from Carl Roth GmbH & Co. KG (Karlsruhe, Germany). β-Lactoglobulin (≥85% purity), 2,5-dihydroxybenzoic acid (2,5-DHB), dioctyl-sulfosuccinate sodium salt (DONS), hydrogen peroxide, N-acetyl-L-cysteine (NAC), *o*-phthalaldehyde (OPA), potassium chloride, pronase E (from Streptomyces griseus, 4,000,000 PU/g), trypsin (from porcine pancreas, 13,000–20,000 BAEE units/mg protein), and polyoxyethylene sorbitan monolaurate (Tween^®^ 20) were obtained from Merck KGaA (Darmstadt, Germany). Pyridine was obtained from Fisher Scientific GmbH (Schwerte, Germany). The peptide calibration standard II for MALDI-TOF-MS analysis was purchased from Bruker Daltonik GmbH (Bremen, Germany). Purified water was obtained from a water purification system with bacteria <1 CFU/10 mL and bacterial endotoxin <0.001 EU/mL (ELGA LabWater, Veolia Water Technologies Deutschland GmbH, Celle, Germany). Unless otherwise specified, ACS-grade was used for all reagents.

#### 2.1.2. Preparation of Cheese Samples

The cheese samples of Edam type were prepared in one of the pilot plants (Milk-Innovation Center (MIC) at Edewecht, Germany) of Deutsches Milchkontor GmbH (DMK), Germany and the Department of Safety and Quality of Milk and Fish Products, Max Rubner Institute (MRI), Kiel, Germany, according to the procedure described by Hoffmann et al. (2019) [52]. On the one hand, control cheeses were made purely from pasteurized milk (0% high-heated milk—0% HH milk). On the other hand, 10%, 20%, or 30% (*w*/*w*) HH milk was mixed with pasteurized milk and used as cheese milk for the production of three different whey protein-enriched Edam cheeses. The high-temperature treatment was carried out at 95 °C for 120 s.

For sample set I, each production line (10%, 20%, and 30% HH milk) had a reference cheese (0% HH milk) made from the same raw milk. The samples were collected at different stages of the cheese manufacturing process. These included the pasteurized and the cheese milk, a whey mixture, and cheese samples before and after the salt bath and after one to six weeks of ripening. The whey mixture is composed of whey obtained at three different times of the cheese-making process and was added together according to their mass balances (*w*/*w*/*w*).

At one of DMK’s MIC, four different and separate approaches under industrial conditions were undertaken in an up-scaled procedure (sample set II). Cheese samples with 0% and 30% HH milk as well as with 10% and 20% HH milk were produced from the same batch of raw milk, respectively.

Table 1 gives an overview of the samples analyzed in this study, including the number of sample replicates per proportion of HH milk in the cheese milk and the different stages of production at which the samples were taken. In addition, the two cheese production processes differed in the performance and number of pasteurizations. In the case of sample set I, the high-heating of the milk was carried out without prior pasteurization of the raw milk. After mixing the pasteurized milk (72–73 °C, 18 s) and the HH milk, no further pasteurization was carried out before the cheese-making process. For sample set II, HH milk was produced from milk that had already been pasteurized (74.5 °C, 15–30 s). The respective mixture of pasteurized and HH milk was pasteurized again (74.0 °C, 15–30 s) before cheese making.

#### 2.1.3. Antibodies

Polyclonal primary antibodies raised in rabbits and directed against human and bovine β-LG were purchased from GeneTex Inc. (Irvine, CA, USA). The antibodies were dissolved in phosphate-buffered saline (PBS) at a concentration of 1 mg/mL. As secondary antibodies, polyclonal goat anti-rabbit antibodies labeled with horseradish peroxidase (HRP) (0.25 mg/mL) were used (Dako GmbH, Glostrup, Denmark).

#### 2.1.4. HPTLC Plates

HPTLC silica gel 60 plates (20 × 10 cm) were purchased from Merck KGaA (Darmstadt, Germany).

### 2.2. Methods

#### 2.2.1. Sample Preparation

##### Protein Extraction

Two slightly different protein extractions were performed. According to a method described by Pellegrino and Tirelli (2000), in the one method, 1 g of each liquid sample or of each shredded cheese sample was mixed with 5 mL of 1 M NaCl and then homogenized for 3 min each using a disperser (Ultra-Turrax^®^) (*n* = 2 for sample set II) [53]. The suspensions were then centrifuged (3220× *g*, 20 min, 4 °C), and 1.5 mL as well as 0.5 mL of the resulting supernatants were lyophilized and subsequently subjected to enzymatic digestion using trypsin (*n* = 2 for sample set II) and pronase E (*n* = 1 for sample set II), respectively. Another 0.5 mL of the supernatant was used for the quantification of amino groups by means of an *o*-phthalaldehyde assay (OPA assay, c.f. Section 2.2.3) (*n* = 1 for sample set II). In the other method, 1 g of the same samples was homogenized with only 4 mL of the 1 M NaCl solution, and 80 µL of this suspension was directly used for tryptic hydrolysis (*n* = 1 for sample set I, *n* = 2 for sample set II). The method involving enzymatic digestion from the lyophilized supernatants is hereinafter referred to as the “supernatant method”, and the method involving direct hydrolysis of the suspensions is referred to as the “suspension method”.

##### Hydrolysis by Trypsin

The lyophilizates of the 1.5 mL supernatants and the 80 µL of the suspensions (c.f. Protein Extraction) were hydrolyzed with trypsin according to Giansanti et al. (2016) [54]. For the suspension method, the volume of solvent (2M urea and 50 mM ammonium bicarbonate in water) was reduced correspondingly by 80 µL; otherwise, the digestion was performed identically for both sample preparations. As a reference standard for the HPTLC analyses, 2 mg of a lyophilized β-LG standard was also digested under the same conditions. In brief, after treatment with DTT and IAA, samples were incubated with 26.6 µL of 0.1% trypsin solution in 50 mM ammonium bicarbonate in water (*w*/*v*) for 12 h at 37 °C. This was followed by a cleanup of the samples by solid-phase extraction using Sep-Pak^®^ C18 cartridges (Waters GmbH, Eschborn, Germany). Conditioning of the cartridges was performed using 100% acetonitrile. Equilibration and washing were carried out with 0.6% acetic acid in water, and elution of the peptides was achieved by 80% (*v*/*v*) acetonitrile and 0.6% (*v*/*v*) acetic acid in water. The eluates were lyophilized and then re-dissolved in 167 µL of the elution buffer and subjected to HPTLC-IS and HPTLC-FS analysis (*n* = 2 for sample set II and supernatant and suspension method; *n* = 1 for sample set I and suspension method) (c.f. Section 2.2.2) and the OPA assay (*n* = 1 for sample set II and supernatant and suspension method) (c.f. Section 2.2.).

##### Hydrolysis by Pronase E

Pronase E digestion was performed according to Kühn et al. (2018) and Krell et al. (2021) [55,56]. A total of 2 mL of a PBS buffer (pH 7.4) was added to the lyophilizates of the 0.5 mL supernatants (c.f. Protein Extraction). Subsequently, pronase E solution (1 mg/mL in PBS buffer, protein:enzyme ratio 100:1) was added, and the mixture was incubated for 18 h at 37 °C on a thermoshaker at 400 rpm. The protein content in the sample extracts of the supernatant method was estimated by the OPA assay (c.f. Section 2.2.3). Hydrolysis was stopped by the addition of 5 µL TFA (≥99%), and the resulting suspensions were centrifuged (3220× *g*, 10 min, 4 °C) afterwards. The obtained supernatants were analyzed by the OPA assay (*n* = 1 for sample set II and supernatant method) (c.f. Section 2.2.3).

#### 2.2.2. High-Performance Thin-Layer Chromatography (HPTLC)

HPTLC separation and detection procedures were performed according to Morschheuser et al. (2017) [35].

##### Separation of the Tryptic Hydrolysates

First, a pre-wash of the silica gel HPTLC plates with methanol and their activation at 100 °C for 10 min was performed. Subsequently, depending on the detection method, 5 µL for FS or 15 µL for IS of the tryptic sample digests were sprayed onto the HPTLC plates as 6 mm bands using an HPTLC autosampler (ATS4, CAMAG AG, Muttenz, Switzerland). In addition, 2 µL of an intact β-LG solution (1 mg/mL in water) and 10–15 µL of the tryptically hydrolyzed β-LG solution were applied as reference standards. Development was performed in twin-trough chambers with a solvent system consisting of 2-butanol/pyridine/ammonia/purified water (39/34/10/26; *v*/*v*/*v*/*v*) up to a solvent migration distance of 80 mm under room temperature and atmospheric pressure. Residual solvents were evaporated in air overnight.

##### Fluorescamine Staining

Protein- or peptide-specific derivatization was performed by immersing the HPTLC plates in a fluorescamine solution (0.05% in acetone) for 1 s at a speed setting of 1 using the chromatogram immersion device III (CAMAG AG, Muttenz, Switzerland). Following evaporation of the solvent, the analytes were visualized under UV light (254 nm, 366 nm) using a photodocumentation system (TLC Visualizer, CAMAG AG, Muttenz, Switzerland).

##### Immunostaining

Immunostaining was performed following Morschheuser et al. (2017) [35]. All subsequent incubations of the IS procedure were performed on a horizontal shaker at 100 rpm and room temperature. The developed and evaporated HPTLC plates were first shaken twice for 15 min each in a blocking reagent (buffer A) consisting of 0.5% (*w*/*v*) Tween^®^ 20, 0.9% (*w*/*v*) sodium chloride, and 0.6% (*w*/*v*) Tris in water. This was followed by incubation with the primary antibody solution (100 μL primary antibody solution in 70 mL buffer A) for 2 h. Thereafter, washing was performed twice for 5 min each with buffer A, followed by a one-hour incubation with the secondary antibody solution (93.3 μL secondary antibody solution in 70 mL buffer A). The HPTLC plates were washed again twice with buffer A for 5 min each before the pH was lowered by a one-minute incubation with 0.06% (*w*/*v*) Tris-HCl in water. Dyeing was performed with a staining reagent composed of two staining solutions: 15 mL of a freshly prepared staining solution 1 (0.24% (*w*/*v*) TMB and 0.80% (*w*/*v*) DONS in ethanol) was added to 45 mL of staining solution 2 (0.94% (*w*/*v*) citric acid monohydrate and 1.5% (*w*/*v*) disodium hydrogen phosphate in water). Immediately prior to coloration, 30 µL of hydrogen peroxide was added to the staining reagent and shaken until the turbidity cleared. At last, the HPTLC plates were incubated in the final staining reagent until blue bands became visible (approximately 15 min). The plates were then dried under room temperature conditions and detected under white light using the TLC visualizer. In order to compare the bands colored by IS, they were quantified by the image processing software Image J (developed at the National Institutes of Health, United States of America, open source) using the integrated density function. The integrated density describes the sum of the values of the pixels in a selection (here: the peptide band) and is defined as the product of the mean gray value and the pixel number. The mean gray value corresponds to the average intensity of the units in the selection [57].

#### 2.2.3. o-Phthalaldehyde Assay (OPA Assay)

The OPA assay for quantification of α- and ε-amino groups was performed according to Aswad (1984) [58]. The samples were diluted differently for the OPA assay depending on their preparation: the non-hydrolyzed samples were diluted with 1 M NaCl to three different ratios, each between 1:1 and 1:50. The samples digested by trypsin and pronase E were also diluted three times each with a 50 mM potassium carbonate buffer at ratios between 1:5 and 1:200. The OPA reagent was prepared by dissolving 25 mg OPA and 58.25 mg NAC in 500 μL ethanol. The solution was then added to 50 mL of the 50 mM potassium carbonate buffer. For external calibration, eight isoleucine or methionine standard solutions were prepared in 50 mM potassium carbonate buffer with concentrations ranging from 1.34 to 67.0 mg/L or 1.28 to 115 mg/L, respectively. Photometric measurement was performed using a microplate reader (Synergy^®^ HT, BioTek, Winooski, VT, USA). A total of 100 µL of the OPA reagent were pipetted into the wells of a microtiter plate, and 100 µL of the sample dilutions or of the isoleucine standard solutions or a blank were added. After shaking the microtiter plates for 2 min, photometric measurement was carried out at 340 nm.

#### 2.2.4. Identification of Amino Acid Sequences by MALDI-TOF-MS/MS

Mass spectrometric identification of the amino acid sequences of selected peptide bands was performed according to a recently described protocol [33]. Briefly, the fluorescamine-derivatized peptide bands of tryptically digested β-LG (*n* = 1) were scraped out from the plate with a scalpel, and the peptides were re-solvated with a solution of 60% (*v*/*v*) acetonitrile and 0.1% (*v*/*v*) formic acid in water. After centrifugation (1700× *g*, 10 min, 4 °C), the supernatants were filtered (regenerated cellulose, 0.45 µm, 25 mm) and concentrated. 2,5-DHB was used as matrix for analysis by MALDI-TOF-MS/MS (ultrafleXtreme™, Bruker Daltonik GmbH, Bremen, Germany). Measurement was performed in positive reflector mode with a mass range of *m*/*z* 340–4000 for MS spectra. The LIFT™ technique was used for MS/MS spectrometry with a mass range of *m*/*z* 10–2500. Amino acid sequences were determined via database research (UniProtKB database in combination with the peptide cutter ExPASy and the PROTEOMICS TOOLKIT Fragment Ion Calculator (Institute for Systems Biology, Seattle, WA, USA).

## 3. Results and Discussion

The stability of β-LG epitopes with regard to a transformation during cheese ripening and a reproducible IS throughout the whole process (from the milk until the final cheese) is a requirement for the validity of the HPTLC-IS method to quantify the whey protein content in the cheese samples. Therefore, the extent to which β-LG still provides antigenicity as intact epitopes after tryptic digestion should be checked primarily. Peptides that are stable during ripening are highly suited as potential process markers for the analysis of whey protein enrichment. Thereby, samples taken at variable times of ripening could be compared with regard to determining the whey protein content. At the hand of such stable process markers, a determination of the extent of the accumulation of whey proteins in the cheese and a correlation with the initial HH milk content would be possible.

### 3.1. Proteolysis of β-Lactoglobulin during the Ripening of Whey Protein-Enriched Cheese

**Figure 1:**
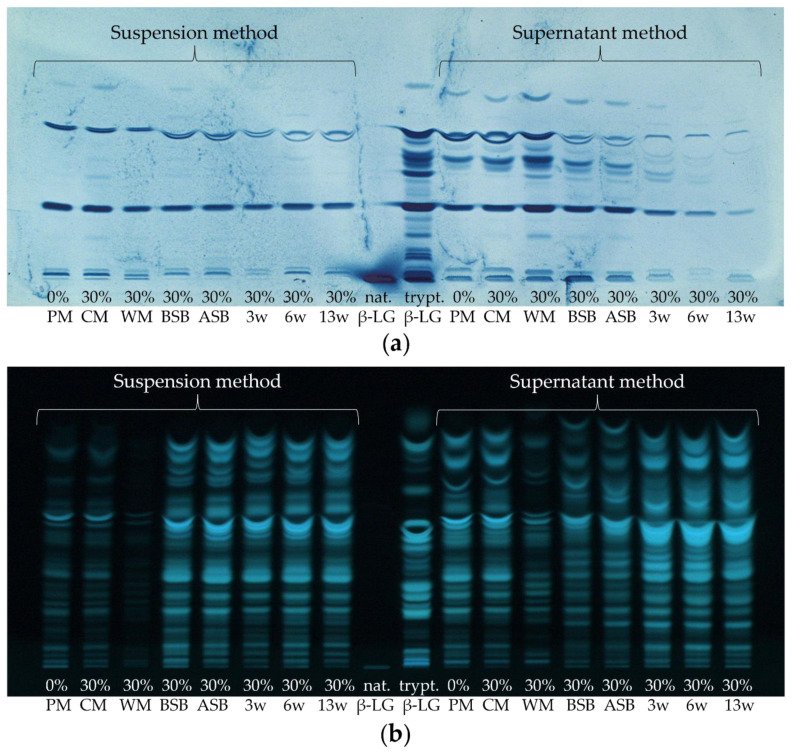
(**a**) HPTLC-IS and (**b**) HPTLC-FS of sample set II of various cheese production stages, containing 30% high-heated milk (HH milk) and of native and tryptically hydrolyzed β-lactoglobulin (nat. and trypt. β-LG). The tryptic hydrolysates of both extraction methods (suspension method or supernatant method) were compared; PM: pasteurized milk, CM: cheese milk (pasteurized milk containing 30% HH milk), WM: whey mixture, BSB: cheese before salt bath, ASB: cheese after salt bath, 3 w: cheese after three weeks of ripening, 6 w: cheese after six weeks of ripening, 13 w: cheese after 13 weeks of ripening.

First, the extent to which β-LG and its corresponding peptides are stable to the manufacturing process and to proteolysis of ripening needs to be evaluated. Figure 1 shows exemplarily one HPTLC-FS and one HPTLC-IS chromatogram of the tryptically hydrolyzed cheese samples of sample set II in the course of the manufacturing process.

The non-hydrolyzed β-LG was applied as a control to verify that the IS detection procedure succeeded (Figure 1a.). Although only a comparatively small volume of non-hydrolyzed β-LG solution (2 µL, 1 mg/mL) was applied to the HPTLC plates, the protein band turned very intensely blue, and the detection procedure could be considered as successful. In contrast, only weak staining of the β-LG band (also 2 µL, 1 mg/mL) was obtained by FS (Figure 1b.). This means that IS with antibodies directed against β-LG detects intact β-LG more sensitively than FS. This is due to the fact that IS is a more specific method compared to FS, which is based on the recognition of epitopes against which the polyclonal antibodies are directed. After HPTLC separation, the undigested β-LG still remains in its native form, allowing both structural and linear epitopes to be bound by the polyclonal antibodies [35]. This is not the case with the tryptically hydrolyzed samples, as the proteins denature when digestion is carried out. This results in a restructuring of the protein and thus a loss of structural epitopes [35,59]. For comparison, Morschheuser et al. (2017) determined a limit of detection of 61.89 ng for the analysis of non-hydrolyzed β-LG by HPTLC-IS. The amount applied here was 2 µg, which corresponds even to approximately the 32-fold of the detection limit determined by Morschheuser et al. [35]. Thus, it is not surprising that the band detected herein has a very intense blue coloration.

When comparing the results of the tryptic hydrolysis of β-LG, 20 bands were detected by IS, while 23 bands were detected by FS. This means that a large proportion of the peptides obtained by tryptic hydrolysis still possess at least one (linear) epitope that leads to the binding of the primary β-LG antibodies. Thus, it can be concluded that the HPTLC-IS method is also suitable for the analysis of tryptically hydrolyzed samples. Morschheuser et al. (2017) were only able to stain four peptides by HPTLC-IS and detected only 15 peptides by HPTLC-FS. Using the UniProtKB database in combination with the peptide cutter ExPASy, the possible peptides theoretically generated by tryptic degradation without any miscleavages were predicted. Considering peptides consisting of at least three amino acids resulted in a number of 15 possible peptides. This would fit very well with the 15 tryptic β-LG peptides detected via FS by Morschheuser et al. (2017) [35]. However, in the present study, up to 23 peptide bands could be detected by HPTLC-FS for tryptically digested β-LG, which could be due to miscleavages or self-digested trypsin [60]. Then, non-tryptic or semi-tryptic peptides may also be formed in which one or more amino acids are truncated at one or both ends (N-terminus and/or C-terminus) of the peptide [61]. In addition, previous experiments have demonstrated (data not shown) that some synthetic β-LG peptide standards also showed multiple bands after HPTLC-IS or HPTLC-FS separation. The appearance of multiple bands could possibly be caused by the formation of dipeptides resulting from newly formed disulfide bridges in the protein or the presence of impurities in the peptide standards [62]. All described phenomena could then lead to a higher number of bands on the HPTLC-FS plate than calculated via the UniProtKB database and ExPASy.

**Figure 2:**
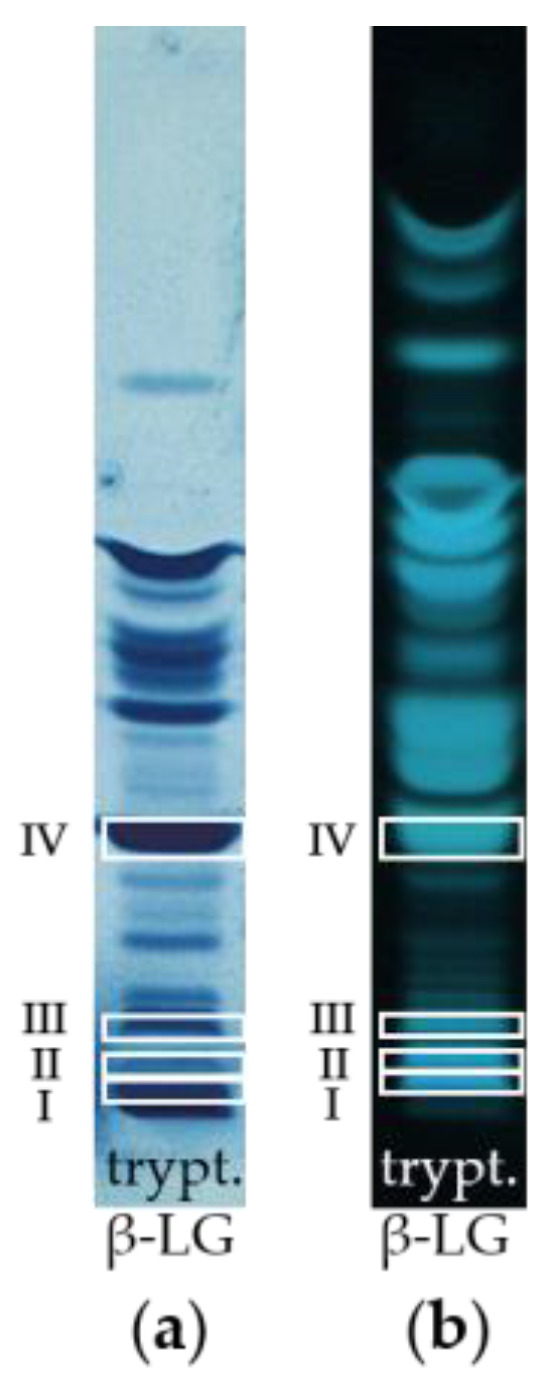
Identified peptide bands of tryptic β-LG by MALDI-TOF-MS/MS after scraping from the HPTLC-FS plate. (**a**) Detection by IS. (**b**) Detection by FS.

In a follow-up approach, it was aimed at identifying the amino acid sequences of the peptide bands from the tryptically hydrolyzed β-LG, which were detected by HPTLC-IS. Thereby, it was possible to determine the amino acid sequence of four peptides (I, II, III, IV) by MALDI-TOF-MS/MS. In Figure 2, the identified peptides in both the HPTLC-IS and the HPTLC-FS chromatogram of the tryptically digested β-LG are framed with white boxes. Three of these peptides (I, II, IV) were also detectable by IS in the cheese samples even after 13 weeks of ripening, indicating that they do not lose their epitope during the proteolysis (c.f. Figure 1a; 13 w).

**Figure 3:**
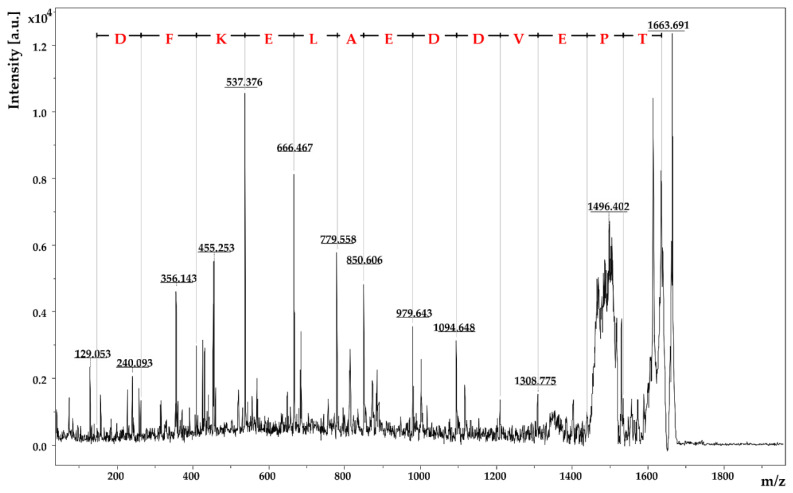
MALDI-TOF-MS/MS spectrum of the parent ion *m*/*z* 1663.69. The amino acid sequence TPEVDDEALEKFDK was identified by the characteristic y- and b-fragments.

Figure 3 shows exemplarily the fragment mass spectrum of the parent ion *m*/*z* 1663.69. Via the mass differences between the signals of the y- and b-fragments of this peptide, the individual amino acids of the primary amino acid sequence could be identified: Peptide I was identified as TPEVDDEALEKFDK (*m*/*z* 1663.69). The other three peptides II–IV are listed in Table 2. Among the four peptides, only one is a full-tryptic peptide without a miscleavage, two peptides show one miscleavage, and one is even a semi-tryptic peptide. Thereby, full-tryptic peptides are the peptides that follow the trypsin cleavage rules. This means that the peptide bond is hydrolyzed adjacent to the basic residues arginine and lysine unless these amino acids are followed by proline [61]. In contrast, a miscleavage would mean that trypsin has failed to hydrolyze a cleavable bond [63]. In semi-tryptic peptides, part of the amino acid sequence is truncated at either the N-terminal or C-terminal end, leaving this peptide with only one trypsin cleavage site [61]. Thereby, semi-tryptic peptides are often formed, depending on the conditions of the digestion [61].

The MALDI-TOF-MS/MS results showed that peptide band IV was the semi-tryptic peptide VYVEELKPTP. However, it cannot be completely excluded that truncation of the complete peptide VYVEELKPTPEGDLEILLQK occurs during the mass spectrometric measurement. In the amino acid sequence of the peptide, two proline residues follow each other very closely, which could lead to an increased risk of special fragmentation [65]. This assumption is also strengthened by the fact that the synthetic reference peptide VYVEELKPTPEGDLEILLQK showed the same retardation factor as the peptide IV (R_f_ value = 0.25) in the HPTLC-FS measurements (data not shown). In addition, spiking of a cheese sample (six weeks ripening, 30% HH milk) with the peptide VYVEELKPTPEGDLEILLQK directly on the HPTLC plate did not result in a separation of the bands (data not shown). When calculating the probable position of the two peptides on the HPTLC plate on the basis of their amino acid composition, or more precisely on the basis of their percentage of non-polar amino acids, an R_f_ value of 0.33 could be obtained for the complete peptide VYVEELKPTPEGDLEILLQK and an R_f_ value of 0.36 for the semi-tryptic peptide VYVEELKPTP [33]. Both calculated R_f_ values are higher than the measured R_f_ value of 0.25. As these two peptides have a similar polarity (the complete peptide contains 45% non-polar amino acids, and the semi-tryptic peptide has 50% non-polar amino acids in their amino acid sequences), they probably differ only slightly in their R_f_ values after separation by HPTLC. Thus, for a complete clarification of the amino acid sequence of the investigated peptide band, a peptide standard of the semi-tryptic peptide VYVEELKPTP would also have to be analyzed. At this stage, based on the mass spectrometric results, it is reasonable to assume that peptide IV is the semi-tryptic peptide VYVEELKPTP.

The four peptides (I–IV) identified by MALDI-TOF-MS/MS are expected to have at least one specific epitope that can bind β-LG antibodies, despite tryptic hydrolysis. The presence and intactness of β-LG epitopes have also been described in the literature for these four peptides (or part of their amino acid sequences):

Parts of the amino acid sequence from the peptides TPEVDDEALEKFDK and TPEVDDEALEK serving as epitopes have been already described by Williams et al. (1998), Miller et al. (1999), Järvinen et al. (2001), Niemi et al. (2007), and Li et al. (2015) [66,67,68,69,70]. Three of these studies also described an epitope activity of the peptide III with its sequence TKIPAVFK [67,68,70]. Furthermore, sequences of the semi-tryptic peptide VYVEELKPTP (peptide IV) were also identified as potential epitopes by Miller et al. (1999), Järvinen et al. (2001), and Niemi et al. (2007) [67,68,69].

When comparing the samples stained with the two detection methods, the first noticeable aspect is that the two different extraction methods resulted in different peptide patterns and intensities (Figure 1). After both detection with IS and FS, the supernatant method showed higher band intensities than the suspension method. However, for the former method, 1.5 mL supernatant of 5 mL extractant was used, and for the latter method, only 0.080 mL of the 5 mL suspension (1 g sample + 4 mL solvent) was used for digestion. If the extraction power and the tryptic digestion are initially assumed to be the same, the peptide bands of the first method should be more intense by a factor of 18.8. However, this is clearly not the case, so this provides a first indication of a poorer extraction strength of the supernatant method. Furthermore, the number of peptide bands detected by IS was also higher for the supernatant method than for the suspension method. On the one hand, this may be due to the higher amount of lyophilizate used for digestion as compared to the suspension, so that possibly the detection limit was exceeded only for the first method. Another reason could be the extraction of different peptides/proteins or possible differences in the enzymatic generation of the peptides with the two extraction methods. Moreover, the milk samples (pasteurized milk (PM) and cheese milk (CM)) and the whey mixture (WM) showed much lower intensities and numbers of peptide bands on the HPTLC-FS plate when applying the suspension method than with the supernatant method. This is again probably due to the insufficient peptide concentration in the sample hydrolysates from the suspension method. However, on the HPTLC-IS plate, these samples showed excellent visible bands with both extraction methods, but the number of bands remained higher for the supernatant method. The high intensity of the bands on the HPTLC-IS plate again suggests that the detection limit by IS is much lower than using FS.

In the following, the individual samples taken during the cheese manufacturing process will be considered for both extraction and detection methods: For example, the whey mixture separated during cheese production showed quite different results in the intensity of the peptide bands. While it showed the highest intensity of all samples on the HPTLC-IS plate for the supernatant method, it showed the lowest intensity of the samples prepared with the suspension method.

The result for the whey mixture obtained by the suspension method was rather as expected, as 30% HH milk was used for the preparation of the samples shown in Figure 1. High-temperature treatment of a part of the CM usually leads to an intensified incorporation of whey proteins into the cheese matrix by denaturing β-LG [1,10]. Thus, a smaller proportion of the whey proteins should run off with the whey. However, not all whey proteins denature to the same extent by high-temperature heating. In addition, temperature and duration of the heating process have an influence on the retention of the individual whey proteins in the cheese [14,71]. The washing of the cheese curd leads to further dilution of the whey so that the protein content in the whey mixture should have by far the lowest protein concentration of all samples. The whey mixture also exhibited the lowest intensities of all samples on the HPTLC-FS plate for both extraction methods. For FS, which is universally used for detecting all primary amino groups, it is not surprising that the whey mixture showed the lowest intensity. This is due to the fact that the protein concentration in whey is only 0.6–1.1%, while a ripened Edam cheese contains about 25% protein [51,72,73]. This underlines that β-LG accounts for a large proportion of the low protein content in the whey mixture, as a strong coloration was obtained by IS anyway. However, the more intense coloration of the WM on the HPTLC-IS plate when analyzing the supernatant compared to the analysis of the suspension does not correspond to the expected lower β-LG content.

Using IS, medium to high intensities were determined with both extraction methods for the two milk samples (PM, CM) compared to the other samples. The intensities of the milk peptide bands even exceeded those of the cheese samples. However, different results were again found on the HPTLC-FS plate. Using the suspension method, higher band intensities were obtained for the milk samples than for the whey mixture, but all cheese samples showed significantly higher intensities than the milk samples. For the supernatant method, the band intensities of the milk samples were not only higher than for the whey mixture but also higher than for the unripened cheese samples (cheese before salt bath (BSB) and after salt bath (ASB)). The FS results also indicate a higher extraction power for the suspension method than for the supernatant method, as bovine milk has a protein content of approximately 3.3% and cheese, as already described, of 25%. Thus, the FS band intensities for the suspension method are consistent with the expected protein concentrations in the samples [72,74]. Both the PM and the CM containing 30% HH milk showed similar intensities to each other on both plates. The reason for this is that even though the high-temperature treatment of the milk leads to the denaturation of the whey proteins, the protein content in the milk samples remains the same. The tryptic digestion led to denaturation of the proteins anyway, so that no relevant difference in intensity between the milk samples was to be expected, as generally only linear epitopes in tryptic peptides are detected by IS.

Comparing the cheese samples obtained by the supernatant method in the course of the ripening, the intensities of the bands decrease visually with time for IS, while the intensities of the peptide bands increase for FS. In contrast, neither an increase nor a decrease is observed in the suspension method during the cheese ripening with both detection methods. As the samples used here are foil-ripened cheeses, the protein concentrations in the cheeses should remain relatively stable during ripening, which again is more consistent with the results of the suspension method. [51].

**Figure 4:**
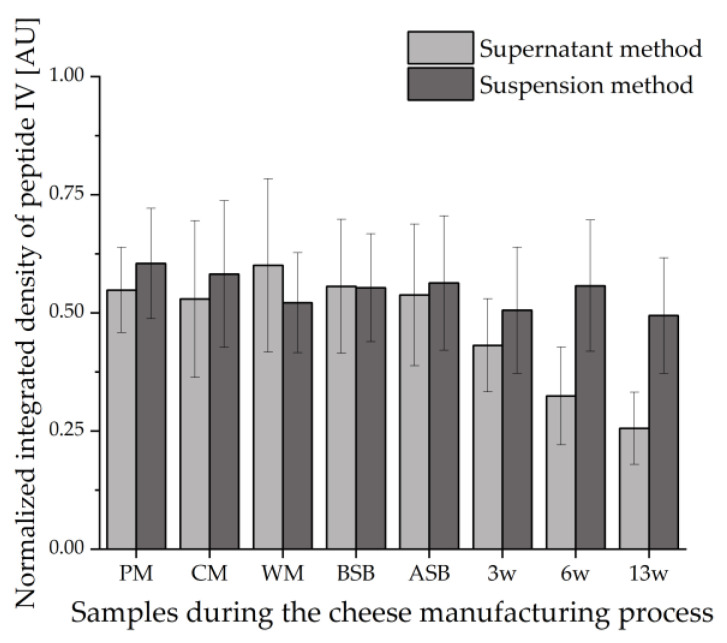
Integrated densities of peptide IV (VYVEELKPTP) normalized to the integrated density of β-LG detected by HPTLC-IS in all samples during the cheese manufacturing process (*n* = 2). The supernatant method was compared with the suspension method; PM: pasteurized milk, CM: cheese milk (pasteurized milk containing 30% HH), WM: whey mixture, BSB: cheese before salt bath, ASB: cheese after salt bath, 3 w: cheese after three weeks of ripening, 6 w: cheese after six weeks of ripening, 13 w: cheese after 13 weeks of ripening.

Figure 4 shows the values of the integrated densities of the bands of peptide IV (VYVEELKPTP) detected by IS for all samples during the cheese manufacturing process. The integrated densities of the peptide bands were determined using the image processing software ImageJ. To ensure comparability between the different HPTLC-IS plates, the values obtained for the integrated densities of the samples were divided by the integrated density of the same peptide band of the tryptically hydrolyzed β-LG standard.

This evaluation also reveals that peptide IV loses intensity during ripening when evaluating with the supernatant method. This suggests that the proteolysis during ripening leads to the degradation of the epitopes that would bind to the primary β-LG antibodies during IS. This effect can also be found in the number of bands detected: On the HPTLC-FS plate, the samples showed no decrease in the number of peptide bands in the course of ripening. In contrast, 13 IS bands were still detectable for the unripened cheese samples BSB and ASB, and only 7 IS bands were detected for the 13-weeks ripened cheese (13 w). On the contrary, the samples obtained by the suspension method showed no trend within the course of ripening, but there is some variation in the values. However, these fluctuations can be considered negligible with respect to the standard deviations (error bars in Figure 4) of the duplicate measurements. This, in turn, means that with the suspension method, it must be presumed that peptides stained by IS remain stable during ripening and are not affected by proteolysis. Looking at the number of detected bands, the peptide pattern does not clearly change between the different samples during the cheese manufacturing process. In all samples, five peptide bands are clearly stained by IS; in some samples, more peptides can be estimated.

For both extraction methods, peptide IV appears to be representative of the intensities in the different samples for the other peptides as well. Peptides I and II were also evaluated using ImageJ via the integrated density and showed similar results (data not shown).

**Figure 5:**
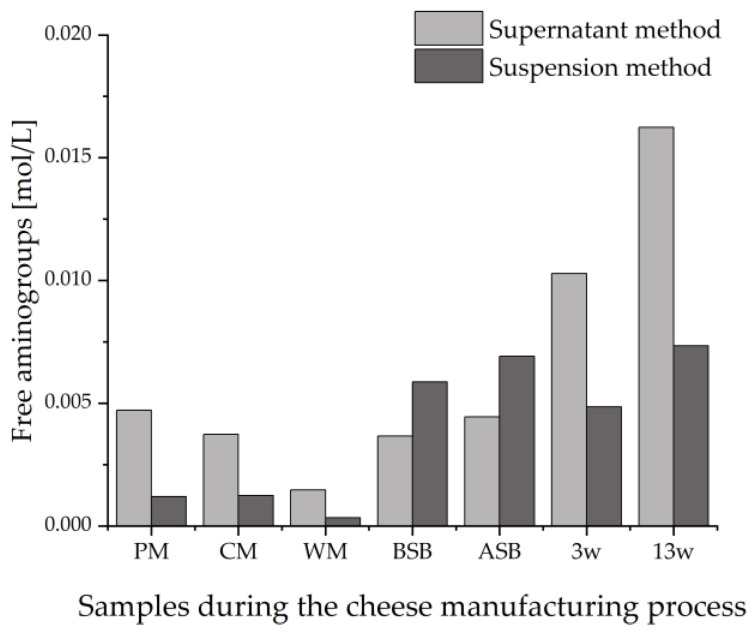
Concentration of free amino groups measured by the OPA assay of tryptically digested samples taken during the cheese production process. The supernatant method was compared with the suspension method; PM: pasteurized milk, CM: cheese milk (pasteurized milk containing 30% HH milk), WM: whey mixture, BSB: cheese before salt bath, ASB: cheese after salt bath, 3 w: cheese after three weeks of ripening, 13 w: cheese after 13 weeks of ripening.

In addition to the intensities of the bands on the HPTLC plates, the protein concentrations in the sample extracts should also be taken into account. For this purpose, the tryptic sample hydrolysates of both extraction methods were analyzed by the OPA assay. Figure 5 shows the different concentrations of free α- and ε-amino groups in the two alternative sample preparations.

The results of the OPA assay of the tryptic peptide extracts were in agreement with the detected intensities of the peptide bands on the HPTLC-FS plate. In contrast to the strong increase in amino groups with ripening in the sample extracts of the supernatant method, there is a decrease in the intensity of the IS bands. As previously suggested, this could be due to a certain degradation of the epitopes resulting from the proteolysis during cheese ripening. For the suspension method, there seems to be no trend in the content of free amino groups in the sample extracts in the course of ripening, just as for the intensities of the bands on the HPTLC-IS/FS plates.

To clarify the conflicting results for the supernatant method, both the undigested sample extracts and the samples hydrolyzed by pronase E were analyzed by the OPA assay. The ratio of the results for the undigested and pronase E digested sample extracts was intended to clarify what type, or more precisely size, of peptides/proteins was extracted from the samples. Digestion with pronase E ideally leads to the complete digestion of proteins and peptides down to their amino acids.

A high ratio of amino groups between the samples digested with pronase E and the undigested samples indicated the presence of larger peptides/proteins in the samples. This can be explained by the fact that in the undigested samples, only the amino groups are measured that are not bound in the peptides and proteins. Only by pronase E digestion do the peptide bonds become hydrolyzed and the amino groups become measurable.

**Figure 6:**
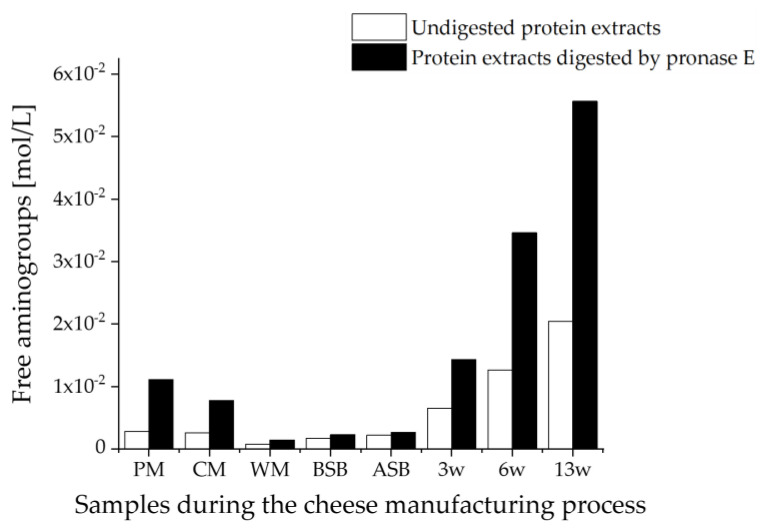
Concentration of free amino groups measured by the OPA assay in the undigested and with pronase E digested samples taken during the cheese production process. The samples were prepared by the supernatant method; PM: pasteurized milk, CM: cheese milk (pasteurized milk containing 30% HH milk), WM: whey mixture, BSB: cheese before salt bath, ASB: cheese after salt bath, 3 w: cheese after three weeks of ripening, 6 w: cheese after six weeks of ripening, 13 w: cheese after 13 weeks of ripening.

Figure 6 shows the concentrations of free amino groups in the two alternative sample preparations for the supernatant method. The undigested samples, as well as the samples digested by pronase E, showed a very similar trend for the free amino group concentrations in the samples and fit well with the OPA results for the tryptic hydrolysates. As expected, the concentrations in the samples digested with pronase E are always higher than in the undigested samples, which is due to the hydrolysis by pronase E to the individual amino acids and thus to the release of many free amino groups. However, the ratios of the contents of amino groups for the undigested and with pronase E digested samples differ within the different samples.

The highest ratios for the free amino group contents between the pronase E digested and the undigested samples were obtained for the two milk samples, with 3.9:1 for the PM and 3.0:1 for the CM. This means that in the milk samples, the largest peptides or proteins were obtained by the supernatant method. However, for the CM containing 30% HH milk, a smaller ratio was obtained than for the entirely pasteurized milk. In contrast, the smallest ratios were determined to be 1.9:1 in the WM, 1.3:1 in the BSB, and 1.2:1 in the ASB. With ripening, the ratio of free amino group contents between the samples digested with pronase E and the undigested samples then increased again, so that a ratio of 2.2:1 was calculated for the 3 w cheese sample and a ratio of 2.7:1 for each of the cheese samples 6 w and 13 w. These results suggest that the supernatant method extracts mostly free peptides and non-denatured proteins and peptides. The WM mainly contains whey proteins, which have smaller molecular weights than caseins [71]. In the cheese samples, β-LG is mainly present as a denatured protein because of the high-temperature treatment. It is bound to the κ-casein by disulfide bridges or generally to the caseins by hydrophobic or electrostatic interactions [1]. As a result, the peptides and proteins might not be well-accessible for extraction with the sodium chloride solution. With cheese ripening, proteolysis of caseins occurs, leading to the release of the peptides that can then be extracted as a result [18]. Dumpler et al. (2017) also described that high-temperature heating of the milk leads to the formation of colloidal casein submicelles to which the whey proteins are bound. These heat-dissociated proteins are less soluble in the extractant and are separated by centrifugation [75]. This also might explain why the suspension method seems to be apparently more suitable for the quantitative extraction of whey proteins from cheese: By digesting the suspensions, not only the whey proteins and peptides dissolved in the extractant but also the proteins/peptides bound to the caseins are subjected to tryptic hydrolysis. The tryptic digestion yields a larger number of unbound peptides, in which water solubility increases due to an increasing polarity. The only disadvantage of this method is that a suspension never contains exactly the same ratio of solids to liquids, which can also be seen in the variations between individual cheese samples and is reflected in a poorer reproducibility (c.f. Figure 4).

**Figure 7:**
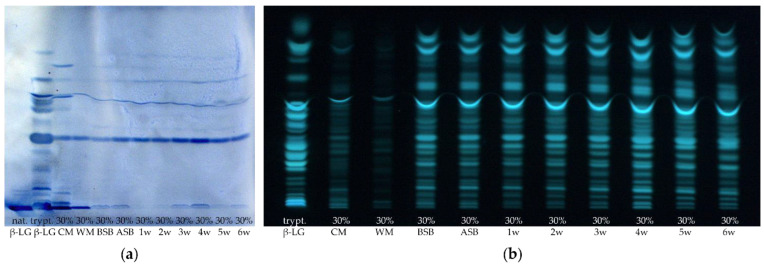
(**a**) HPTLC-IS and (**b**) HPTLC-FS of sample set I of various cheese production stages containing 30% high-heated milk (HH milk) and of native and tryptically hydrolyzed β-lactoglobulin (nat. and trypt. β-LG). The samples were prepared by the suspension method; CM: cheese milk (pasteurized milk containing 30% HH milk), WM: whey mixture, BSB: cheese before salt bath, ASB: cheese after salt bath, 1–6 w: cheese after one to six weeks of ripening.

The repeatability of the method should be investigated using samples from another manufacturer with slightly modified cheese-making conditions (c.f. Section 2.1.2). For this purpose, a production line with 30% HH milk of sample set I was processed by the suspension method and also analyzed by HPTLC-IS and HPTLC-FS. The chromatograms obtained are shown in Figure 7.

**Figure 8:**
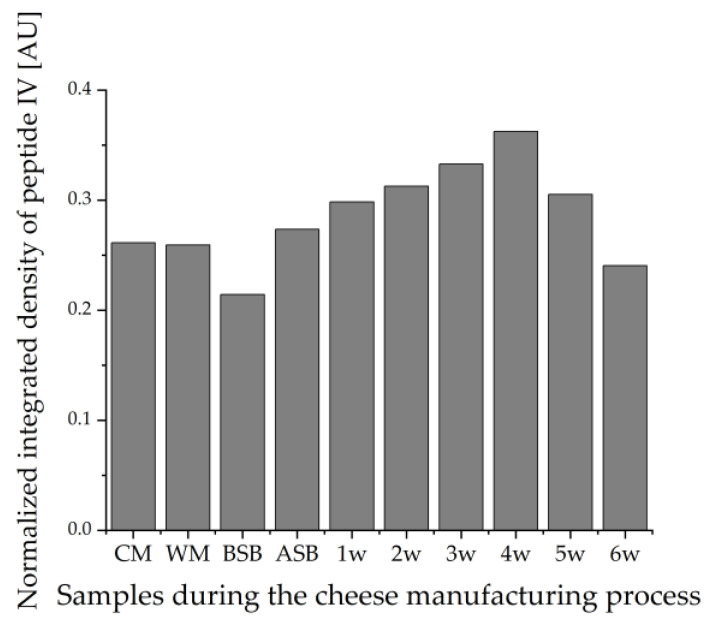
Integrated densities of peptide IV (VYVEELKPTP) normalized to the integrated density of β-LG detected by HPTLC-IS in all samples of a production line of sample set I during the cheese manufacturing process. The samples were prepared by the suspension method; CM: cheese milk (pasteurized milk containing 30% HH milk), WM: whey mixture, BSB: cheese before salt bath, ASB: cheese after salt bath, 1–6 w: cheese after one to six weeks of ripening.

Very similar results were obtained for sample set I as for sample set II. The band intensities on the HPTLC-FS plates again show that the lowest protein concentration is found in the WM, followed by the milk sample. The cheese samples show the highest intensities of all samples, and there appears to be no trend with ripening time either. However, it is noticeable that the cheese sample after 4 weeks of ripening stands out with an increased intensity compared to the other cheese samples. The intensities of the peptides on the HPTLC-IS plate also show that the cheese samples actually have the strongest staining. However, it is visually apparent from this chromatogram that the WM exhibits similar intense staining to the cheese milk and is not lower as in sample set II. This indicates that the WM contains a particularly high proportion of β-LG peptides that possess an epitope. In contrast, the cheese sample BSB shows exceptionally low coloration. For the HPTLC-IS chromatogram, the integrated densities for peptide band IV of the samples were also determined using ImageJ and normalized to the integrated density of the same peptide band of β-LG (Figure 8). The results of the integrated densities match well with the visually detectable band intensities, although the integrated density of the 6-week ripened cheese sample is slightly too low because this band is skewed. The integrated densities of the cheese samples seem to increase until the fourth week of ripening and then decrease again until the sixth week. However, this result should also be interpreted that the β-LG epitopes in the cheese samples remain relatively stable during ripening, and just the individual samples taken after different times of ripening vary from each other. This is also shown by the fact that for both detection methods, the 4-week ripened cheese sample stands out with particularly high band intensities, and thus, the protein content of this sample is probably generally above average.

It can be summarized that different results were obtained for the two different extraction methods regarding the stability of the β-LG epitopes in the tryptic peptides during cheese ripening. For the supernatant method, a decrease in detectable β-LG epitopes could be assumed. However, it was also found that this extraction method is likely to extract mainly free peptides and native peptides and proteins, so the results cannot be considered reliable. In contrast, no trend for β-LG epitopes with ripening time was observed for the suspension method for two different sample sets. This means that the β-LG peptides studied here remain relatively stable during the ripening period and are thus generally well suited as biomarkers for the quantification of the whey protein content in whey protein-enriched cheese.

### 3.2. Enrichment of β-Lactoglobulin in Whey Protein-Enriched Cheese

**Figure 9:**
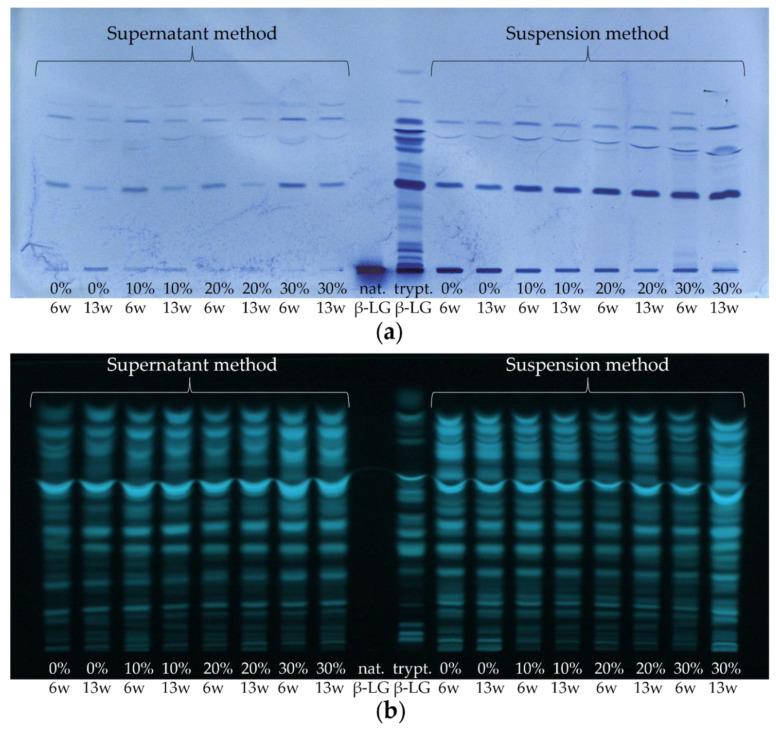
(**a**) HPTLC-IS and (**b**) HPTLC-FS of sample set II and of native or tryptically digested β-LG (nat. β-LG, trypt. β-LG). Cheeses were ripened for 6 and 13 weeks (6 w, 13 w), respectively, containing 0–30% HH milk (0%, 10%, 20%, and 30%). The supernatant method was compared with the suspension method.

For the investigation of whey protein enrichment in whey protein-enriched cheese, four different model cheeses containing 0%, 10%, 20%, or 30% HH milk (sample set II) were analyzed. For this purpose, the study was limited to cheese samples that had ripened for 6 and 13 weeks, respectively, in order to represent products that could also be similar to commercially available products. In Figure 9, one HPTLC-IS and one HPTLC-FS chromatogram of the tryptically hydrolyzed cheese samples are shown as examples. Moreover, the two extraction methods (supernatant method and suspension method) were compared again.

First, looking at the HPTLC-FS plate, it is noticeable that a large number of bands were detected when the cheese samples were stained with FS universally, with some bands even overlapping (c.f. Figure 9b). Thereby, no differences were detectable between samples with different levels of HH milk (0–30%) and between the ripening durations of 6 and 13 weeks. Only the cheese sample ripened for 13 weeks, made with 30% HH milk and prepared by the suspension method, shows comparatively particularly high band intensities on the HPTLC-FS plate. The reason for the non-FS detectable differences between the cheese samples with varying contents of HH milk is probably due to the low content of β-LG (and correspondingly the other whey proteins) in the cheese compared to the caseins. As a result, the casein peptides overlap with the whey protein peptides so that no enrichment can be observed. Thus, by HPTLC-FS, only an approximate estimation of the number and intensity of peptides present in the cheese samples can be obtained, but the protein origin (caseins/whey proteins) of the peptides remains unknown.

In contrast, peptides can be detected very specifically using the HPTLC-IS method, allowing the protein origin to be identified. In this work, the peptides of β-LG were selectively stained in the ripened cheese samples using IS (c.f. Figure 9a). On the HPTLC-IS chromatogram, only a few but very well separated bands were detected. It can already be observed visually that the intensity of the bands increased with the increasing content of HH milk. Thereby, for the supernatant method, the intensities of the peptide bands from the 6 weeks ripened cheese samples are slightly higher than those from the 13 weeks ripened cheese samples. In the suspension method, on the other hand, no difference can be seen on the HPTLC-IS plate between the different ripening times. These two trends are consistent with the results from Section 3.1. For the supernatant method, this indicates a decrease in β-LG epitopes with increasing ripening time, whereas, for the suspension method, stability of β-LG epitopes can be assumed.

**Figure 10:**
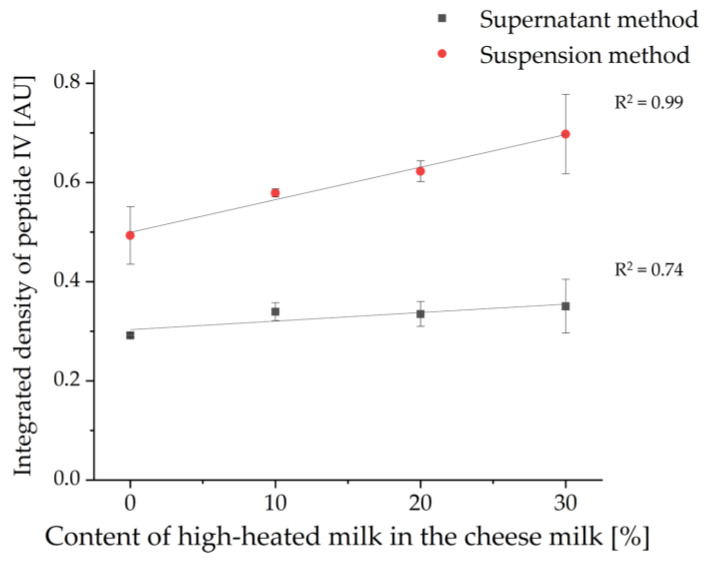
Linear regression of the concentration of tryptic β-LG peptides plotted as normalized integrated density of peptide IV (VYVEELKPTP) in relation to the content of HH milk in the cheese milk used for the production of the 6- and 13-weeks ripened cheeses of sample set II (*n* = 2). Again, the supernatant method was compared with the suspension method.

Nevertheless, in contrast to the HPTLC-FS method, this methodology enables detecting differences between samples with different contents of HH milk. The level of tryptic β-LG peptides increased with the level of HH milk in the CM. For each of the two extraction methods, the integrated density was determined for the band of peptide IV (VYVEELKPTP) and normalized to the integrated density of the same band of β-LG as previously described in Section 3.1. This peptide band was chosen as it showed the highest intensity of all peptides stained by IS and was very well separated from the other peptides. In Figure 10, the values obtained for the integrated density were plotted against the initially HH milk content added for both extraction methods. For this, the values for the integrated densities of the 6- and 13-weeks ripened cheese samples were averaged. As already visually recognizable, the determination of the integrated density also showed a positive correlation between the concentration of the tryptic β-LG peptides and the content of HH milk in the cheese samples (c.f. Figure 9a). The relationship was found to be linear, with R^2^ values of 0.74 for the supernatant method of 0.99 for the suspension method. However, for both methods, one of the two HPTLC-IS measurements yielded a value that was too low for the 13-week ripened cheese made with 30% HH milk and was not included in the calculation as an outlier. The worse R^2^ value of 0.74 for the supernatant method could be due to averaging of the values obtained for the 6- and 13-weeks ripened cheeses on the one hand and to the incomplete extraction of denatured peptides and proteins on the other hand (c.f. Section 3.1).

To determine an enrichment factor for the accumulation of β-LG in the cheese samples studied, the integrated density of peptide IV (VYVEELKPTP) of the cheese samples made with 30% HH milk was divided by the integrated density of the same peptide of the cheese samples made with 0% HH milk. Unfortunately, with both extraction methods, one of the two HPTLC-IF measurements for the cheese ripened for 13 weeks showed an integrated density value that was too low for the cheese with 30% HH milk. As outliers, these two values were not included in the calculation of the enrichment factor, but the values for the integrated density of the cheese containing 20% HH milk were used instead [14]. Enrichment factors of 1.24 ± 0.11 were determined for the supernatant method and of 1.33 ± 0.19 for the suspension method. Interestingly, the enrichment factors differ only slightly between the two extraction methods. This is likely due to the fact that similarly aged samples were compared, so the influence of the poorer extraction power of the supernatant method is small. The reason for this may be that all samples, regardless of the percentage of HH milk used to prepare the cheese samples, ripened similarly.

For comparison, Guinee et al. (1995) determined enrichment factors of 1.81 to 4.63 for the whey proteins in three differently produced whey protein-enriched cheeses.

However, as different amounts of the cheese milk were high-heated and the heat treatment conditions (temperature, time) differed in the cheese production processes (Guinee et al. vs. the sample sets I and II used here), the results are not properly comparable. For example, Guinee et al. determined a denaturation rate of 51.5% of the whey proteins for cheese with the highest heat treatment of the milk and in the cheeses studied in the present study, only up to 30% of the milk was high-heated at all [14].

The relative enrichments of β-LG of 24% or 33% determined in the present study, using HPTLC-IS, refers only to the behavior of peptide IV (VYVEELKPTP). Its rates cannot be generalized to all peptides generated from β-LG by tryptic digestion because the various peptides may be affected differently during ripening by proteolysis. As mentioned above, proteolysis can occur endogenously from the initial milk, the enzyme preparations added for casein denaturation, starter culture bacteria, or ubiquitously cultures such as mold and non-lactic acid bacteria [15,17,21]. Consequently, the release of certain peptides might differ. Moreover, VYVEELKPTP is a semi-tryptic peptide, so the degree of hydrolysis by trypsin in addition to ripening is unknown. Indeed, it is not known in what ratio the semi-tryptic peptide VYVEELKPTP is formed compared to the full-tryptic peptide VYVEELKPTPEGDLEILLQK during tryptic hydrolysis. However, the enrichment factor can only be applied to β-LG and not to all whey proteins, as, due to the handling procedures, different proportions of whey proteins can remain in the cheese [10,11].

Nevertheless, in order to obtain an estimation of the relative content at least of β-LG (C_β-LG%_) in the cheese samples studied, the following Equation (1) was used for calculation for both extraction methods. Here, m_β-LG_ or m_sample_ correspond to the mass of β-LG used for tryptic digestion or the mass of the weighed sample, ID_sample_ or ID_β-LG_ stands for the integrated density of peptide IV of the samples or of β-LG, respectively, and DF for dilution factor. The dilution factor for the supernatant method is 3.33, and for the suspension method, it is 62.5.

C_β-LG%_ = (m_β-LG_ × ID_sample_ × DF × 100%)/(m_sample_ × ID_β-LG_) (1)

Very different results were obtained with this calculation for the relative content of β-LG in the cheese samples for the two extraction methods. Using the supernatant method, a relative β-LG content of 0.25% ± 0.09% was determined in cheeses made with 30% HH milk. On the other hand, using the suspension method, the relative β-LG content was determined to be 9.11% ± 0.29%, which is 36 times higher than with the other method. This again makes it very clear how much more quantitative the suspension method is versus the supernatant method. If the determined 9.11% β-LG in the entire cheese is related to protein content in Edam of approximately 25%, about one-third of the total protein of the cheese produced with 30% HH milk consists of β-LG. This seems to be a rather high value if one considers that only 30% of the milk was high-heated and that the caseins in the initial milk are four times more concentrated than the whey proteins. The increased value is probably due to the fact that only a one-point calibration with a tryptically hydrolyzed β-LG standard was performed and the intensity of the band of peptide IV was much higher with the tryptically hydrolyzed β-LG than with the cheese samples. For a more accurate result, a calibration curve should be used. Either tryptically digested β-LG could continue to be used as the standard, or a synthetic peptide standard could be employed.

Peptide IV (VYVEELKPTP) might have the potential to serve as a process marker. In practice, cheese samples could be compared using the HPTLC-IS method to determine the enrichment factor of peptide IV and thus infer the content of the HH milk used in cheese production. The relative content of β-LG in the cheese can also be calculated via a calibration curve. However, this does not yet clarify the extent to which the β-LG content can be extrapolated back to the total whey protein content. In addition, a targeted LC-MS/MS approach to quantify peptide IV could also be used to determine a whey protein enrichment factor or the relative content of β-LG in cheese samples.

**Figure 11:**
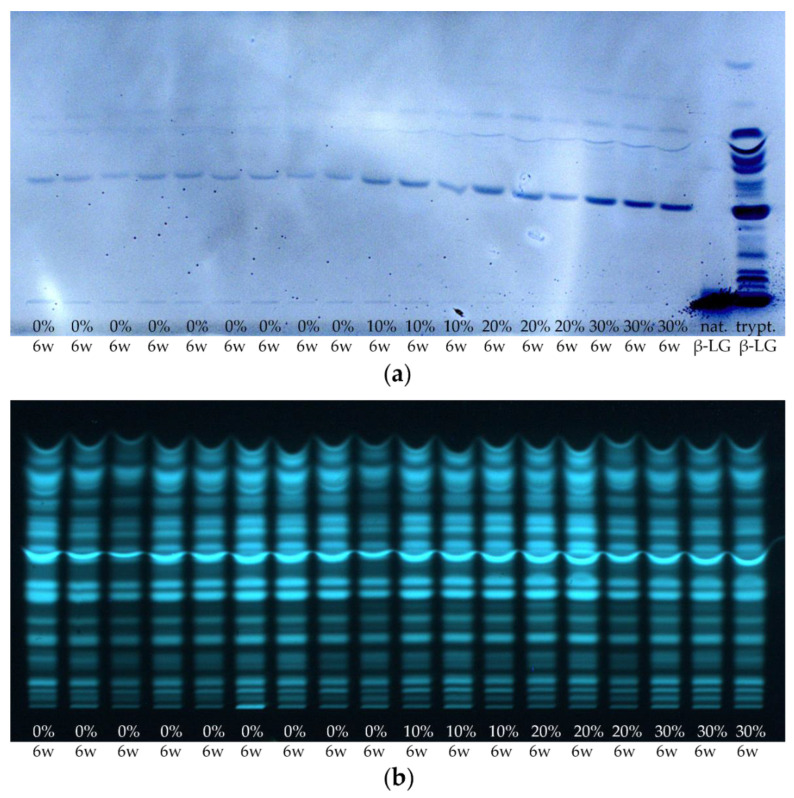
(**a**) HPTLC-IS and (**b**) HPTLC-FS of the cheese samples of sample set I and of native or tryptically digested β-LG (nat. β-LG, trypt. β-LG). Cheeses were ripened for 6 weeks (6 w), containing 0–30% HH milk (0%, 10%, 20%, and 30%). The samples were prepared by the suspension method.

As in Section 3.1, it is now also to be checked to what extent the results are reproducible for other samples from different manufacturers. For this reason, the 6 weeks ripened cheese samples of all 18 cheese production lines of sample set I were processed by the suspension method and analyzed by HPTLC-IS and HPTLC-FS (Figure 11).

As already found for the industrial-scale DMK Edam cheese samples, an increase in band intensities can be visually observed on the HPTLC-IS plate with increasing content of HH milk used for the production of the different cheese samples. In contrast, no trend can be detected on the HPTLC-FS plate in terms of band intensities related to the content of HH milk used.

**Figure 12:**
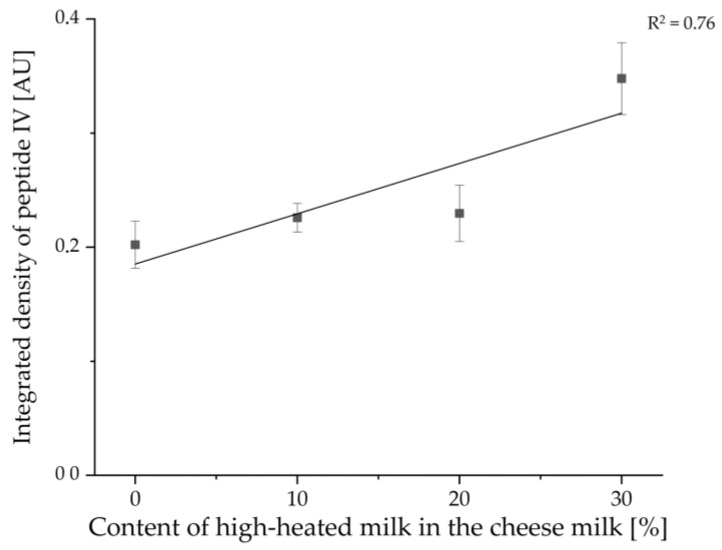
Linear regression of the concentration of tryptic β-LG peptides plotted as normalized integrated density of peptide IV (VYVEELKPTP) in relation to the content of HH milk in the cheese milk used for the production of the different cheeses of sample set I. The samples were prepared by the suspension method.

With the help of the software ImageJ, the integrated densities of the different samples for peptide IV were also determined for the measurement of sample set I. Figure 12 shows the results for the average normalized integrated densities against the content of HH milk used for the production of the cheeses. Two outliers were not included in the calculation: In each case, the third sample with 10% or 20% HH milk applied to the HPTLC-IS plate showed values that were very low. In the case of the cheese sample with 10% HH milk, this is due to an uneven band shape, and in the case of the sample with 20% HH milk, this is due to the light background coloration at that position on the HPTLC-IS plate (c.f. Figure 11a). Furthermore, only an R^2^ value of 0.76 was obtained for the linear regression. In Figure 12, it can be seen that the cheese samples made with 20% HH milk had very low values for the integrated density. The enrichment factor was calculated to be 1.72, which is higher than the values for sample set II. However, both samples sets are in the same range, and only one HPTLC-IS measurement was performed for sample set I, where the value for the cheese with 30% HH milk is slightly above the calibration curve (c.f. Figure 12).

The mean relative content of β-LG determined via Equation (1) in the cheese samples of sample set I prepared with 30% HH milk is 4.35% ± 0.39%. Thus, only about half was detected with the same extraction method in sample set II. However, the value of 4.35% or about one-sixth of the total protein in the cheese samples seems more realistic. As only an estimation of the content via a single-point calibration was performed for both sample sets, the quantification of the β-LG content in cheese requires further research.

## 4. Conclusions

Using HPTLC-IS, it was not only possible to stain intact β-LG very sensitively, but even to detect 20 bands of β-LG peptides, indicating the presence of β-LG epitopes in these peptides. This demonstrated that it is generally possible to apply HPTLC-IS for the analysis of β-LG peptides in tryptically hydrolyzed samples.

Different results were obtained when comparing the two different extraction methods and the two detection methods in relation to the cheese manufacturing process: it was found that the supernatant method is not quantitative. In contrast, the suspension method resulted in a quantitative extraction of proteins/peptides from the cheeses. Thereby, the β-LG epitopes proved to be stable toward proteolysis with increasing ripening time, which is why these peptides seem to be appropriate as potential process markers for the quantification of whey protein content in cheese. In addition, the comparison of ripened cheese samples produced with different levels of HH milk in the cheese milk showed that only the HPTLC-IS method, which is selective for β-LG (and its peptides), was able to detect the accumulation of whey proteins in the cheese samples.

The positive relationship between the accumulation of the semi-tryptic peptide VYVEELKPTP and the content of HH milk in the cheese samples was found to be linear. Thereby, similar whey protein enrichment factors of 1.24 ± 0.11 and 1.33 ± 0.19 were determined between the cheese samples containing 30% HH milk and 0% HH milk for the supernatant method and the suspension method, respectively. Nevertheless, the method presented herein can only be used to determine a factor estimating the enrichment between two samples. In order to be able to calculate the absolute or relative content of whey proteins in any cheese samples, a factor is missing by which the content of whey proteins can be determined from the concentration of a specific tryptic β-LG peptide. However, this requires the assumption that the whey protein composition is kind of uniform in the different cheese samples and types.

A one-point calibration with the tryptically hydrolyzed β-LG allowed at least an estimation of the relative concentration of β-LG in the cheese samples. For the supernatant method, the insufficient extraction power was again evident, while for the suspension method, a value of 9.11% ± 0.29% was determined.

In addition, the method was demonstrated to be applicable to cheese samples from other manufacturers and with slightly different manufacturing protocols. Here, a whey protein enrichment factor of 1.72 was determined between the samples with 30% and 0% HH milk, and the estimated content of β-LG in the cheese samples obtained was 4.35% ± 0.39%.

Based on the results obtained, it is not possible to emphasize which of the slightly different procedures used to prepare the two sample sets resulted in a higher enrichment of whey protein in the cheese. In fact, a higher enrichment factor was calculated for sample set I, but a lower relative content of β-LG was observed in those cheese samples.

The advantages of the HPTLC-IS method are the specific detection of β-LG peptides, which can be used to quantify the whey protein content in cheese. However, so far, only the β-LG content has been primarily taken into account, and a calibration curve with at least five calibration points should be used for more accurate quantification. In order to infer the whey protein content from the marker peptide, further experiments need to be performed to determine a conversion factor. In addition, it should be considered that inconsistent background staining of the HPTLC-IS plates may cause inaccurate results.

Nevertheless, it can be finalized that the HPTLC-IS method presented here is a promising method for the quantification of the whey protein content in cheeses. Therefore, it bears the potential for being a promising and valuable tool for industrial implementation.

## Figures and Tables

**Table 1:** Overview of the analyzed cheese samples from sample set I and sample set II; PM: pasteurized milk, CM: cheese milk (pasteurized milk containing 30% HH milk), WM: whey mixture, BSB: cheese before salt bath, ASB: cheese after salt bath.

Sample Set	HH Milk [%]	*n*	Samples of Different Cheese Production Stages			
			Milk Samples	Whey Samples	Cheese Curd Samples	Ripening Length of Cheese Samples [Weeks]
I	0	9	-	-	-	6
	10	3	-	-	-	6
	20	3	-	-	-	6
	30	3	CM (n = 1)	WM (n = 1)	BSB, ASB (n = 1)	1–6
II	0	1	PM	-	-	6, 13
	10	1	-	-	-	6, 13
	20	1	-	-	-	6, 13
	30	1	CM	WM	BSB, ASB	3, 6, 13

**Table 2:** Peptides containing at least one epitope that binds antibodies directed against β-LG identified by MALDI-TOF-MS/MS. The Roman numbers correspond to those in Figure 2 and throughout the main text [64].

Peptide	Rf Value	*m*/*z* (MS1)	Position	Amino Acid Sequence	Miscleavages	(M + H)^+^[u]	Mass Difference [u]	Adducts
I	0.03	1663.69	141–154	TPEVDDEALEKFDK	1	1635.77	28	dimethylation
II	0.05	1537.55	141–151	TPEVDDEALEK	0	1245.58	292 (260 + 32)	fluorescamine + methanol
III	0.09	1163.57	92–99	TKIPAVFK	1	903.57	260	fluorescamine
IV	0.25	1198.62	57–66	VYVEELKPTP	semi-tryptic peptide	1156.62 (of the b-fragment)	42	trimethylation

## References

[1] Masotti F., Cattaneo S., Stuknytė M., De Noni I. (2017). Technological tools to include whey proteins in cheese: Current status and perspectives. Trends Food Sci. Technol..

[2] Abd El-Gawad M.A.M., Ahmed N.S. (2011). Cheese yield as affected by some parameters review. Acta Sci. Pol. Technol. Aliment..

[3] Mulvihill D.M., Donovan M. (1987). Whey proteins and their thermal denaturation—A review. Irish J. Food Sci. Technol..

[4] Smithers G.W. (2008). Whey and whey proteins—From ‘gutter-to-gold’. Int. Dairy J..

[5] Wagner J., Biliaderis C.G., Moschakis T. (2020). Whey proteins: Musings on denaturation, aggregate formation and gelation. Crit. Rev. Food Sci. Nutr..

[6] Schingoethe D.J. (1976). Whey Utilization in Animal Feeding: A Summary and Evaluation. J. Dairy Sci..

[7] Le Maux S., Bouhallab S., Giblin L., Brodkorb A., Croguennec T. (2014). Bovine β-lactoglobulin/fatty acid complexes: Binding, structural, and biological properties. Dairy Sci. Technol..

[8] Edwards P.J.B., Jameson G.B. (2020). Structure and Stability of Whey Proteins.

[9] Petit J., Herbig A., Moreau A., Delaplace G. (2011). Influence of calcium on ß-lactoglobulin denaturation kinetics: Implications in unfolding and aggreagtion mechanisms. J. Dairy Sci..

[10] Hinrichs J. (2001). Incorporation of whey proteins in cheese. Int. Dairy J..

[11] Lelievre J. (1995). Whey proteins in cheese—An overview. Adv. Exp. Med. Biol..

[12] Chromik C., Partschefeld C., Jaros D., Henle T., Rohm H. (2010). Adjustment of vat milk treatment to optimize whey protein transfer into semi-hard cheese: A case study. J. Food Eng..

[13] Hickey C.D., Auty M.A.E., Wilkinson M.G., Sheehan J.J. (2015). The influence of cheese manufacture parameters on cheese microstructure, microbial localisation and their interactions during ripening: A review. Trends Food Sci. Technol..

[14] Guinee T.P., Pudja P.D., Reville W.J., Harrington D., Mulholland E.O., Cotter M., Cogan T.M. (1995). Composition, microstructure and maturation of semi-hard cheeses from high protein ultrafiltered milk retentates with different levels of denatured whey protein. Int. Dairy J..

[15] Donato L., Guyomarc’h F. (2009). Formation and properties of the whey protein/κ-casein complexes in heated skim milk—A review. Dairy Sci. Technol..

[16] Van den Berg G., Exterkate F.A. (1993). Technological parameters involved in cheese ripening. Int. Dairy J..

[17] Niero G., Sturaro A., Trentin A.R., Masi A., De Marchi M., Cassandro M. (2014). Effect of cheesemaking with microparticulated whey proteins on the concentration of low molecular thiols in cheese. Acta Agrar. Kaposváriensis.

[18] Ardö Y., McSweeney P.L.H., Magboul A.A.A., Upadhyay V.K., Fox P.F. (2017). Biochemistry of Cheese Ripening: Proteolysis.

[19] Creamer L.K., Zoerb H.F., Olson N.F., Richardson T. (1982). Surface Hydrophobicity of αs1 -I, αs1 -Casein A and B and Its Implications in Cheese Structure. J. Dairy Sci..

[20] Afshari R., Pillidge C.J., Dias D.A., Osborn A.M., Gill H. (2020). Cheesomics: The future pathway to understanding cheese flavour and quality. Crit. Rev. Food Sci. Nutr..

[21] Martini S., Conte A., Tagliazucchi D. (2020). Effect of ripening and in vitro digestion on the evolution and fate of bioactive peptides in Parmigiano-Reggiano cheese. Int. Dairy J..

[22] Abd El-Salam M.H. (2014). Application of proteomics to the areas of milk production, processing and quality control—A review. Int. J. Dairy Technol..

[23] Enright E., Patricia Bland A., Needs E.C., Kelly A.L. (1999). Proteolysis and physicochemical changes in milk on storage as affected by UHT treatment, plasmin activity and KIO3 addition. Int. Dairy J..

[24] Gagnaire V., Piot M., Camier B., Vissers J.P.C., Jan G., Léonil J. (2004). Survey of bacterial proteins released in cheese: A proteomic approach. Int. J. Food Microbiol..

[25] Pappa E.C., Robertson J.A., Rigby N.M., Mellon F., Kandarakis I., Mills E.N.C. (2008). Application of proteomic techniques to protein and peptide profiling of Teleme cheese made from different types of milk. Int. Dairy J..

[26] Molina E., Ramos M., Amigo L. (2002). Characterisation of the casein fraction of Ibérico cheese by electrophoretic techniques. J. Sci. Food Agric..

[27] Gaiaschi A., Beretta B., Poiesi C., Conti A., Giuffrida M.G., Galli C.L., Restani P. (2001). Proteolysis of β-casein as a marker of Grana Padano cheese ripening. J. Dairy Sci..

[28] Mehta-D’Souza P. (2012). Detection of glycoproteins in polyacrylamide gels using pro-q emerald 300 dye, a fluorescent periodate schiff-base stain. Methods Mol. Biol..

[29] Gwarda R.Ł., Dzido T.H. (2013). Two-dimensional high-performance thin-layer chromatography of tryptic bovine albumin digest using normal- and reverse-phase systems with silanized silica stationary phase. J. Chromatogr. A.

[30] Zhang Y., Chen R., Ma H., Chen S. (2015). Isolation and Identification of Dipeptidyl Peptidase IV-Inhibitory Peptides from Trypsin/Chymotrypsin-Treated Goat Milk Casein Hydrolysates by 2D-TLC and LC-MS/MS. J. Agric. Food Chem..

[31] Panchagnula V., Mikulskis A., Song L., Wang Y., Wang M., Knubovets T., Scrivener E., Golenko E., Krull I.S., Schulz M. (2007). Phosphopeptide analysis by directly coupling two-dimensional planar electrochromatography/thin-layer chromatography with matrix-assisted laser desorption/ionization time-of-flight mass spectrometry. J. Chromatogr. A.

[32] Emory J.F., Walworth M.J., Van Berkel G.J., Schulz M., Minarik S. (2010). Direct analysis of reversed-phase high-performance thin layer chromatography separated tryptic protein digests using a liquid microjunction surface sampling probe/electrospray ionization mass spectrometry system. Eur. J. Mass Spectrom..

[33] Treblin M., von Oesen T., Class L.-C., Kuhnen G., Clawin-Rädecker I., Martin D., Fritsche J., Rohn S. (2021). Two-dimensional high-performance thin-layer chromatography for the characterization of milk peptide properties and a prediction of the retention behavior—A proof-of-principle study. J. Chromatogr. A.

[34] Spöttel J., Brockelt J., Badekow S., Rohn S. (2021). Immunological Analysis of Isothiocyanate-Modified α-Lactalbumin Using High-Performance Thin Layer Chromatography. Molecules.

[35] Morschheuser L., Mink K., Horst R., Kallinich C., Rohn S. (2017). Immunological analysis of food proteins using high-performance thin-layer chromatography-immunostaining. J. Chromatogr. A.

[36] Morschheuser L., Wessels H., Pille C., Fischer J., Hünniger T., Fischer M., Paschke-Kratzin A., Rohn S. (2016). HPTLC-aptastaining—Innovative protein detection system for high-performance thin-layer chromatography. Sci. Rep..

[37] Badekow S., Treblin M., Spöttel J., Rohn S. (2021). Benzyl isothiocyanate-modified α-lactalbumin—Two-dimensional high-performance thin-layer chromatography for analyzing modified peptides. J. Chromatogr. B.

[38] Pasilis S.P., Kertesz V., Van Berkel G.J., Schulz M., Schorcht S. (2008). Using HPTLC/DESI-MS for peptide identification in 1D separations of tryptic protein digests. Anal. Bioanal. Chem..

[39] Biller J., Morschheuser L., Riedner M., Rohn S. (2015). Development of optimized mobile phases for protein separation by high performance thin layer chromatography. J. Chromatogr. A.

[40] Morschheuser L., Mükusch S., Riedner M., Seitz H., Rohn S. (2016). High-performance thin-layer chromatography as a fast screening tool for phosphorylated peptides. J. Chromatogr. B.

[41] Gwarda R., Tomczyszyn A., Misicka A., Dzido T.H. (2015). Retention and separation efficiency of some synthetic oligopeptides in reversed-phase thin-layer chromatography. Acta Chromatogr..

[42] Morlock G., Schwack W. (2010). Hyphenations in planar chromatography. J. Chromatogr. A.

[43] Tscherch K., Biller J., Lehmann M., Rohn S. (2013). One- and Two-dimensional High-performance Thin-layer Chromatography as an Alternative Analytical Tool for Investigating Polyphenol—Protein Interactions. Phytochem. Anal..

[44] Bhusan R., Mahesh V.K., Mallikharjun P.V. (1989). Thin layer chromatography of peptides and proteins: A review. Biomed. Chromatogr..

[45] Urbanova I., Svec F. (2011). Monolithic polymer layer with gradient of hydrophobicity for separation of peptides using two-dimensional thin layer chromatography and MALDI-TOF-MS detection. J. Sep. Sci..

[46] Bakry R., Bonn G.K., Mair D., Svec F. (2007). Monolithic porous polymer layer for the separation of peptides and proteins using thin-layer chromatography coupled with MALDI-TOF-MS. Anal. Chem..

[47] Srivastava M. (2011). High-Performance Thin-Layer Chromatography (HPTLC).

[48] Papasotiriou D.G., Markoutsa S., Gorka J., Schleiff E., Karas M., Meyer B. (2013). MALDI analysis of proteins after extraction from dissolvable ethylene glycol diacrylate cross-linked polyacrylamide gels. Electrophoresis.

[49] Puranik M., Bhawsar D.V., Rathi P., Yeole P.G. (2010). Simultaneous Determination of Ofloxacin and Ornidazole in Solid Dosage Form by RP-HPLC and HPTLC Techniques. Indian J. Pharm. Sci..

[50] Loescher C.M., Morton D.W., Razic S., Agatonovic-Kustrin S. (2014). High performance thin layer chromatography (HPTLC) and high performance liquid chromatography (HPLC) for the qualitative and quantitative analysis of *Calendula officinalis*—Advantages and limitations. J. Pharm. Biomed. Anal..

[51] Düsterhöft E.M., Engels W., Huppertz T. (2017). Gouda and Related Cheeses.

[52] Hoffmann W., Luzzi G., Steffens M., Clawin-Rädecker I., Franz C.M.A.P., Fritsche J. (2019). Salt reduction in film-ripened, semihard Edam cheese. Int. J. Dairy Technol..

[53] Pellegrino L., Tirelli A. (2000). A sensitive HPLC method to detect hen’s egg white lysozyme in milk and dairy products. Int. Dairy J..

[54] Giansanti P., Tsiatsiani L., Low T.Y., Heck A.J.R. (2016). Six alternative proteases for mass spectrometry-based proteomics beyond trypsin. Nat. Protoc..

[55] Kühn C., von Oesen T., Hanschen F.S., Rohn S. (2018). Determination of isothiocyanate-protein conjugates in milk and curd after adding garden cress (*Lepidium sativum* L.). Food Res. Int..

[56] Krell M., Cvancar L., Poloczek M., Hanschen F.S., Rohn S. (2021). Determination of Isothiocyanate-Protein Conjugates in a Vegetable-Enriched Bread. Foods.

[57] Seletchi E.D., Duliu O.G. (2007). Image processing and data analysis in computed tomography. Rom. J. Phys..

[58] Aswad D.W. (1984). Determination of d- and l-aspartate in amino acid mixtures by high-performance liquid chromatography after derivatization with a chiral adduct of o-phthaldialdehyde. Anal. Biochem..

[59] Neurath H., Greenstein J.P., Putnam F.W., Erickson J.O. (1944). The chemistry of protein denaturation. Chem. Rev..

[60] Nord F.F., Bier M., Terminiello L. (1956). On the mechanism of enzyme action. LXI. The self digestion of trypsin, calcium-trypsin and acetyltrypsin. Arch. Biochem. Biophys..

[61] Alves P., Arnold R.J., Clemmer D.E., Li Y., Reilly J.P., Sheng Q., Tang H., Xun Z., Zeng R., Radivojac P. (2008). Fast and accurate identification of semi-tryptic peptides in shotgun proteomics. Bioinformatics.

[62] Rahali V., Chobert J.M., Haertlé T., Guéguen J. (2000). Emulsification of chemical and enzymatic hydrolysates of β-lactoglobulin: Characterization of the peptides adsorbed at the interface. Nahr.—Food.

[63] Šlechtová T., Gilar M., Kalíková K., Tesařová E. (2015). Insight into Trypsin Miscleavage: Comparison of Kinetic Constants of Problematic Peptide Sequences. Anal. Chem..

[64] Zhang Y., Fonslow B.R., Shan B., Baek M.-C., Yates J.R. (2013). Protein Analysis by Shotgun/Bottom-up Proteomics. Chem. Rev..

[65] Lottspeich F., Engels J.W. (2018). Bioanalytics.

[66] Williams S.C., Badley R.A., Davis P.J., Puijk W.C., Meloen R.H. (1998). Identification of epitopes within beta lactoglobulin recognised by polyclonal antibodies using phage display and PEPSCAN. J. Immunol. Methods.

[67] Miller K., Meredith C., Selo I., Wal J.M. (1999). Allergy to bovine β-lactoglobulin: Specificity of immunoglobulin E generated in the Brown Norway rat to tryptic and synthetic peptides. Clin. Exp. Allergy.

[68] Järvinen K.M., Chatchatee P., Bardina L., Beyer K., Sampson H.A. (2001). Identification of IgE and IgG binding epitopes on β- and κ-casein in cow’s milk allergic patients. Int. Arch. Allergy Immunol..

[69] Niemi M., Jylhä S., Laukkanen M.L., Söderlund H., Mäkinen-Kiljunen S., Kallio J.M., Hakulinen N., Haahtela T., Takkinen K., Rouvinen J. (2007). Molecular Interactions between a Recombinant IgE Antibody and the β-Lactoglobulin Allergen. Structure.

[70] Li X., Yuan S., He S., Gao J., Chen H. (2015). Identification and characterization of the antigenic site (epitope) on bovine β-lactoglobulin: Common residues in linear and conformational epitopes. J. Sci. Food Agric..

[71] Wong D.W.S., Camirand W.M., Pavlath A.E. (1996). Structures and Functionalities of Milk Proteins. Crit. Rev. Food Sci. Nutr..

[72] Heino A., Uusi-rauva J., Outinen M. (2010). Pre-treatment methods of Edam cheese milk. Effect on cheese yield and quality. LWT—Food Sci. Technol..

[73] Prazeres A.R., Carvalho F., Rivas J. (2012). Cheese whey management: A review. J. Environ. Manag..

[74] Le T.T., Deeth H.C., Larsen L.B. (2016). Proteomics of major bovine milk proteins: Novel insights. Int. Dairy J..

[75] Dumpler J., Wohlschläger H., Kulozik U. (2017). Dissociation and coagulation of caseins and whey proteins in concentrated skim milk heated by direct steam injection. Dairy Sci. Technol..

